# Distinct tumor signatures using deep learning-based characterization of the peritumoral microenvironment in glioblastomas and brain metastases

**DOI:** 10.1038/s41598-021-93804-6

**Published:** 2021-07-14

**Authors:** Zahra Riahi Samani, Drew Parker, Ronald Wolf, Wes Hodges, Steven Brem, Ragini Verma

**Affiliations:** 1 Diffusion and Connectomics in Precision Healthcare Research Lab (DiCIPHR), Department of Radiology, University of Pennsylvania, Philadelphia, PA USA; 2 Department of Radiology, Department of Neurosurgery, University of Pennsylvania, Philadelphia, PA USA; 3 Founder at Synaptive Medical, Toronto, ON Canada

**Keywords:** Cancer imaging, Cancer in the nervous system, Biomedical engineering, Cancer imaging

## Abstract

Tumor types are classically distinguished based on biopsies of the tumor itself, as well as a radiological interpretation using diverse MRI modalities. In the current study, the overarching goal is to demonstrate that primary (glioblastomas) and secondary (brain metastases) malignancies can be differentiated based on the microstructure of the peritumoral region. This is achieved by exploiting the extracellular water differences between vasogenic edema and infiltrative tissue and training a convolutional neural network (CNN) on the Diffusion Tensor Imaging (DTI)-derived free water volume fraction. We obtained 85% accuracy in discriminating extracellular water differences between local patches in the peritumoral area of 66 glioblastomas and 40 metastatic patients in a cross-validation setting. On an independent test cohort consisting of 20 glioblastomas and 10 metastases, we got 93% accuracy in discriminating metastases from glioblastomas using majority voting on patches. This level of accuracy surpasses CNNs trained on other conventional DTI-based measures such as fractional anisotropy (FA) and mean diffusivity (MD), that have been used in other studies. Additionally, the CNN captures the peritumoral heterogeneity better than conventional texture features, including Gabor and radiomic features. Our results demonstrate that the extracellular water content of the peritumoral tissue, as captured by the free water volume fraction, is best able to characterize the differences between infiltrative and vasogenic peritumoral regions, paving the way for its use in classifying and benchmarking peritumoral tissue with varying degrees of infiltration.

## Introduction

Discriminating tumor types is undertaken by neuroradiologists, trained to recognize imaging patterns, and is confirmed by a surgical biopsy of the tumor. There has been growing interest in developing computational methods to distinguish tumor types using diverse MRI modalities^1^, different parts of the tumors^2^, characteristics of the peritumoral region^3^, or a combination of all the aforementioned^4^. In this paper, we exploit the differences in extracellular water content of the peritumoral region to distinguish tumor types, specifically, brain metastases and glioblastomas.

Diffusion Tensor Imaging (DTI) provides an insight into the extracellular water content of tissue. Recent studies have attempted to use DTI to discriminate metastases and glioblastomas^2^. The metrics commonly used include DTI-derived measures of mean diffusivity (MD) and fractional anisotropy (FA)^5^ from both the tumor and peritumoral regions. They applied thresholding on the metrics or performed machine learning techniques^3,6–11^ (e.g., texture analysis^12,13^) to characterize the tumor differences. Multiparametric approaches combining different modalities of MR imaging have also been used to discriminate between brain metastases and glioblastomas^1,4,14,15^, however, using a single modality would reduce the complexity of analysis and total imaging time needed for multiparametric acquisitions.

There are known differences in the microstructural tissue characteristics of brain metastases and glioblastomas^1^. Fundamental to the biology and clinical management of glioblastomas is their propensity to invade the peritumoral tissue, so that the peritumoral area consists of edema but also invasive cells. By contrast, the peritumoral region of metastases consists mainly of acellular fluid^2^. These variations in tissue composition are reflected in DTI-derived measures of FA and MD^2,16^, but would benefit from superior modeling of microstructure that can be achieved by multi-compartment models. FERNET^17^ is a multi-compartment modeling technique that extracts free water volume fractions (FW-VF) representative of extracellular water content from diffusion MR data. FW-VF can aid in exploiting the differences in extracellular water within the peri-tumoral area^17–19^ of infiltrative or vasogenic edema. Our aim is to use this information to differentiate tumor types.

Deep learning tools, especially convolutional neural networks (CNNs), are particularly effective in elucidating local patterns^20,21^. They learn the underlying features of importance as well as the discrimination algorithm. We use CNNs to extract patterns from free water volume fraction maps and capture deep visual features in extracellular water content of brain metastases and glioblastomas and discriminate them.

We compare the performance of the CNN trained on the FW-VF map with the one trained with radiomics and texture features^1^. We also evaluate the performance of free water corrected fractional anisotropy, and axial and radial diffusivity in discriminating tumor type to demonstrate how the distinction performs after removing extracellular water from the peritumoral microenvironment.

This paper proposes a new method based on free water that can differentiate tumor types based on the tissue characteristics of the peritumoral region. The microstructure is captured by learning the patterns of extracellular water content between the infiltrated tissue and vasogenic edema with CNNs. This method is tested on metastases and glioblastomas and performs better than those created using the traditional measures of FA and MD. It can be used in classifying and characterizing peri-tumoral tissue with varying degrees of infiltration.

## Method

### Overview

The aim of this paper is to characterize two major types of brain tumors, metastases and glioblastomas, based on microstructural characteristics of peritumoral edema derived from their DTI-based free water volume fraction (FW-VF) map. We create a 2D CNN-based classifier trained on FW-VF patches located in the peritumoral edema to distinguish brain metastases and glioblastomas. We first describe the patient data and then the details of the CNN-based classifier. The performance of the CNN trained on the FW-VF map is then compared to those trained on: *i*) standard fractional anisotropy (FA), *ii*) mean diffusivity (MD), *iii)* combination of FA and MD, *iv*) free water corrected fractional anisotropy (FW- FA), *v*) axial (FW-AX), *vi*) radial (FW-RAD) diffusivity, and *vii)* combination of FW-FV and FW-FA. Lastly, the CNN is compared with texture and radiomic features.

### Patient data and creation of free water volume fraction (FW-VF)

This study was approved by the institutional review board of University of Pennsylvania. Informed consent was obtained from all participants or their legally authorized representative. All methods were carried out in accordance with relevant guidelines and regulations.

The baseline demographics, clinical and molecular characteristics of the patients were as follows. The training cohort consisted of 106 patients (66 glioblastomas and 40 metastases) with a mean age of 61.1 years ± 12.1 (standard deviation), (range, 23–87 years), including 55 men and 51 women. The most common primary cancer that metastasized was lung cancer (21), followed by melanoma (5), breast (4), and others (10) and we had two patients with multi-focal metastatic lesion. GBM patients were all IDH-1 non-mutants (wild-types), and we had 13 patients with history of prior resection. The test cohort comprised 30 patients, including 20 glioblastomas and 10 patients with metastasis who had a mean age of 64.3 years ± 10.4 (range, 42–84 years), with 16 men and 14 women. The most common primary cancer of metastasis was lung cancer (6), followed by melanoma (2), breast (1), and others (1) and we had one patient with multi-focal metastatic lesion in our test cohort. GBM patients were all IDH-1 non-mutants (wild-types), and we had 3 patients with history of prior resection in our test cohort. There was no significant difference in age between patients with glioblastomas and metastases in either the training or test cohort (p = 0.53, p = 0.14 respectively). Furthermore, there was no significant difference in the proportion of metastases and glioblastomas (p = 0.69) and lung versus non-lung proportion (p = 0.65) of metastases primary cancer between the training and test cohorts.

dMRI/DTI data was acquired on two types of scanners, 118 patients with the Siemens 3 T TrioTim and 18 patients with the Siemens 3 T Verio, both with TR/TE = 5000/86 ms, resolution = 1.72 × 1.72 × 3 mm, 3 b = 0 s/mm2 volumes, and 30 diffusion weighted volumes with b = 1000 s/mm2. The dMRI data was pre-processed using local PCA denoising^22^, eddy current and motion correction performed using FSL EDDY^23^, and skull-stripping with BET^24^. FA and MD maps were computed after DTI fitting with DIPY using weighted least squares^25^.

**Figure 1:**
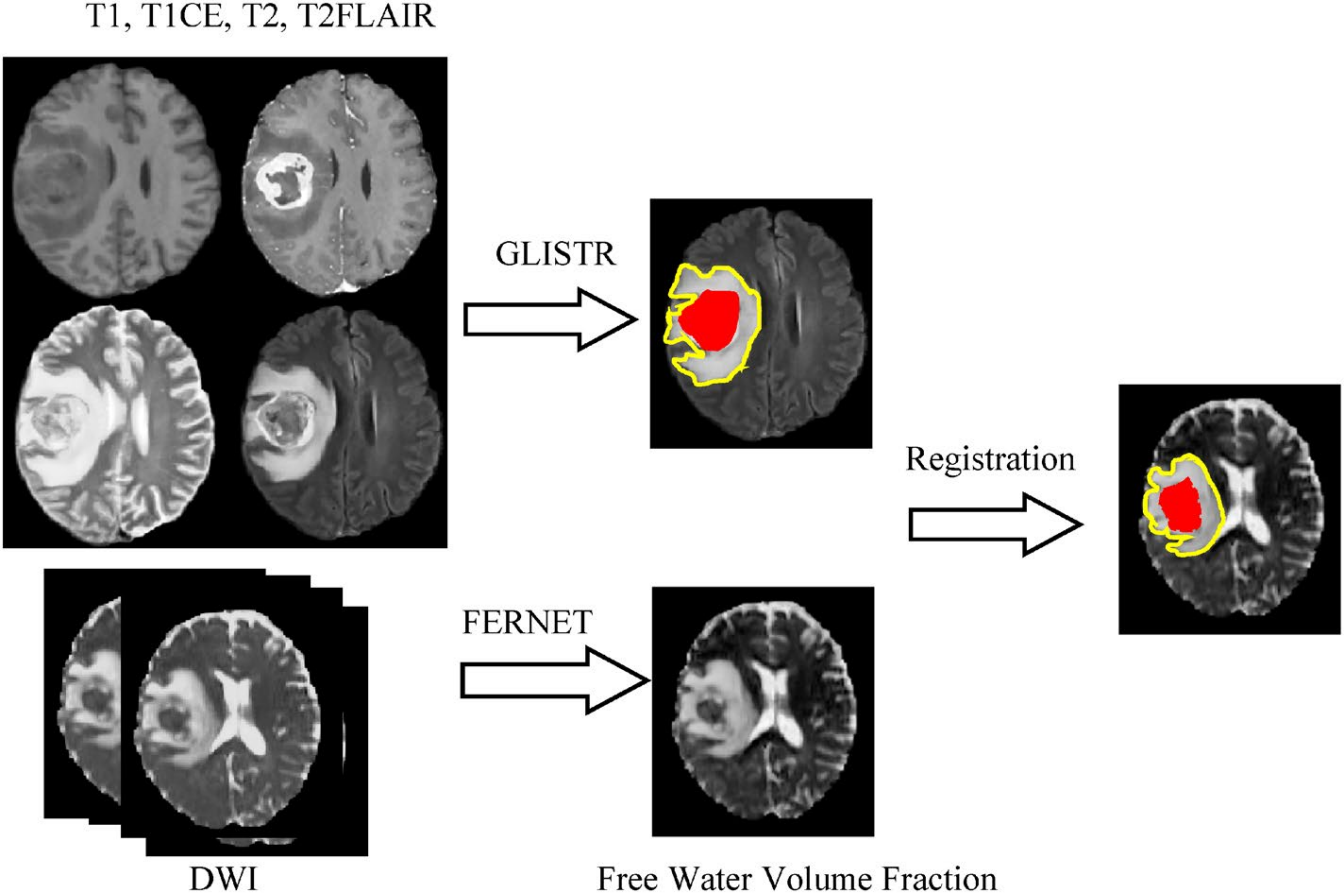
The pre-processing steps of our method. Segmentation was done using GLISTR on structural MRI (T1, T1-CE, T2, T2-FLAIR). Freewater EstimatoR using Interpolated Initialization (FERNET) was used for estimating the free water volume fraction. Mask of tumor and edema were then registered to DTI data.

Masks of the tumor and edema for each patient were created using GLISTR^26,27^, a semi-automated tumor segmentation tool that uses structural data (T1, T1-CE, T2, T2-FLAIR) to make the segmentation. GLISTR produces regions of enhancing, non-enhancing and necrotic tumor, which we combined to form the tumor region, and produces a region of edema, which is hyperintense in FLAIR and T2. It is independent of location and number of tumors. Therefore, in case there were more than one lesion per tumor, GLISTR would produce tumor and edema masks for all lesions. Mask of tumor and edema were then registered to DTI data^26^. Details are presented in Fig. 1.

For estimating the free water volume fraction from single shell DTI, we used Freewater EstimatoR using Interpolated Initialization (FERNET)^17^, a free water elimination paradigm using a novel interpolated initialization approach, that estimates the free water compartment in single-shell diffusion MRI data. FERNET provides a free water volume fraction map (FW-VF), and free water corrected fractional anisotropy (FW-FA), axial diffusivity (FW-AX) and radial diffusivity (FW-RAD), for every patient from their pre-processed dMRI data^28^.

### CNN based classifier using FW-VF for discriminating metastatic tumors from glioblastomas

**Figure 2:**
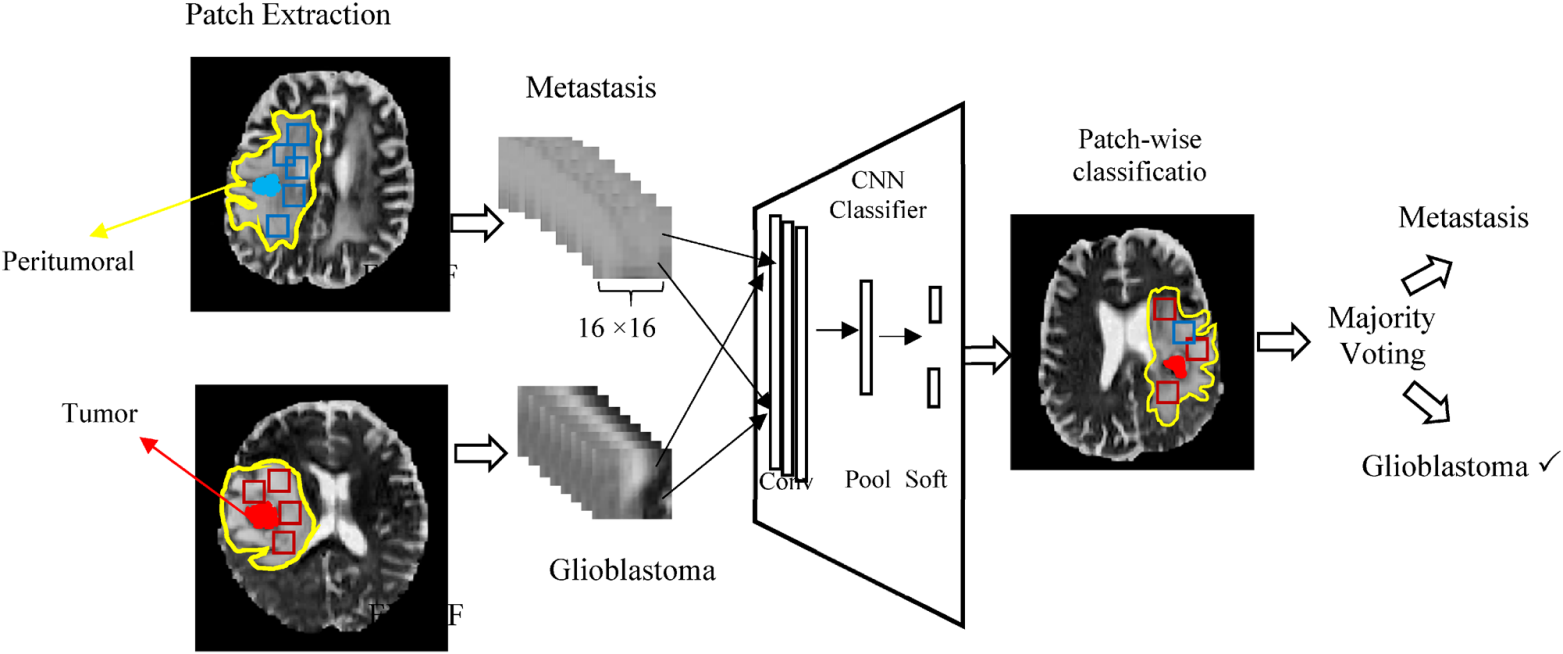
The pipeline of our classifier: input to the classifier were patches (boxes) extracted from the free water volume fraction map in peritumoral area from both glioblastoma (red) and metastases (blue) which were used to train the CNN. In test phase, the results of CNN on patches were combined by majority voting to get the final label of metastasis or glioblastoma for each patient.

We created a CNN classifier trained on patches derived from the peritumoral region to assign a label of metastasis or glioblastoma. Figure 2 shows the pipeline of our approach. We automatically extracted input patches for our CNN in the peritumoral area of metastatic and glioblastoma subjects. Random seed generators were used to choose location of patches. A set of (16 × 16) patches from peritumoral edema was extracted for every subject. This was the largest patch we could fit into peritumoral edema without overlapping into the tumor and was large enough for the CNN classifier to capture specific patterns. We iteratively chose patches in random locations in edema, and toward random coordinates, and discarded patches that included the tumor itself. In the case it was more than one tumor in a patient, we selected patches from the peritumoral areas of all of the tumors. The number of patches in every subject was estimated based on the number of edema voxels in that subject divided by the number of voxels in the patch (16 × 16 = 256). All patches extracted from a subject were assigned the tumor label of the subject, that is, metastasis or glioblastoma.

The classifier was based on convolutional neural networks (CNNs)^29^, which are a special kind of neural network, composed of a set of convolutional and pooling layers in their architecture. Our convolutional (conv) layers were connected to local parts of input patches to detect local features from them. We put 6 convolutional layers followed by pooling (pool) layers that reduced the dimension of the features. We put a max pooling and a global average pooling layer that calculated the average of each feature map and prepared the feature vector for the classification layer. We put a softmax layer (soft) at the end that produced a probability value for every input patch which indicated its membership to each class (metastases or glioblastomas, in our case)^30^. As we trained our classifier on patches with different patterns of extracellular water, this number illustrated the local signature of extracellular water for each patch. The label for each patch was assigned to a class with maximum probability value.

Data augmentation was done on the patches by shifting them in different directions, allowing up to 20% overlap with healthy brain. This was to avoid overfitting of the classifier. We created ~ 6300 patches (~ 3000 metastases and ~ 3300 glioblastomas) from the training subjects. CNN training was done for 100 epochs using the rmsprop optimizer and a cross entropy loss function. More details on CNN hyperparameters are provided in supplementary material S.1.

For testing, a set of patches were automatically extracted in the peritumoral region of test subjects using random seed generators. The number of patches in every subject was estimated based on the number of edema voxels in that subject divided by the number of voxels in the patch (16 × 16 = 256). The CNN classifier assigned labels to all patches and the final label of a subject was calculated using majority voting among the classification on patches.

### Evaluation of the classifier performance

The following measures were used to evaluate the performance of the CNN classifier:

$$Accuracy = \frac{{TP + TN}}{{TP + TN + FP + FN}}$$

$$Sensitivity = \frac{{TP}}{{TP + FN}}$$

$$Specificity = \frac{{TN}}{{TN + FP}}$$

With glioblastoma standing for our positive class, true positive (TP) represents the number of cases correctly recognized as glioblastoma, false positive (FP) represents the number of cases incorrectly recognized as glioblastoma, true negative (TN) represents the number of cases correctly recognized as metastasis and false negative (FN) represents the number of cases incorrectly recognized as metastasis. Thus, sensitivity represents the recall value for glioblastoma class and specificity represent recall the values for metastases class.

#### Cross validation and test results

We evaluated our CNN classifier in 5-fold cross-validation, and a test setting. For cross-validation, the patches from all the trainings were shuffled and randomly partitioned into 5 equally sized subsamples. For each run, a single subsample was retained as validation data while the remaining 4 subsamples were used as training data, and this process was repeated for all 5 subsamples. The reported measures were averaged among all folds which represented the performance of 2D CNN classifier over the patches.

In addition to our cross-validation settings, the CNN classifier was evaluated on a set of 30 independent test subjects that were kept out of the training process. We applied our CNN to all patches in peritumoral area of test subjects and the subject class label was calculated by majority voting among patches.

#### Comparison of efficacy of FW-VF classifier with those created from the other dMRI-derived maps

In order to demonstrate the superiority of FW-VF in discriminating metastases and glioblastomas, we retrained the CNN using patches derived from free-water-corrected fractional anisotropy (FW-FA), axial diffusivity (FW-AX), and radial diffusivity (FW-RAD), as well as the traditional mean diffusivity (MD) and fractional anisotropy (FA) maps. The results were compared based on accuracy, sensitivity, and specificity. We also created combination classifiers (FW-VF map and FW-FA) and compared the performance with the single feature classifiers. A combination classifier was also created for FA and MD maps.

#### Comparison of CNN with radiomic and texture-based classifiers

Finally, we compared our CNN classifier with those trained on traditional texture features and classifiers. We applied Gabor feature extractors and radiomic features^31^ in combination with random forest classifiers. Gabor features were constructed from the response of applying Gabor filters made on several frequencies (scales) and orientations^32^. We applied Gabor filters with 4 directions and 4 scales. Radiomic features included size and shape-based features, descriptors of image intensity histogram, descriptors of the relationships between image voxels, textures extracted from filtered images, and fractal features^33^. We used the PyRadiomics^34^ package to extract radiomic features followed by principal component analysis which reduced feature dimensions to cover 98% of variation in the data.

### Ethics declarations

This study was approved by the institutional review board of University of Pennsylvania. Informed consent was obtained from all participants or their legally authorized representative. All methods were carried out in accordance with relevant guidelines and regulations.

## Results

### Cross-validation and test results

Table 1 shows the cross-validation and test results for our CNN-based classifier with free water volume fraction (FW-VF). We obtained 85% accuracy in our cross-validation result with a sensitivity and specificity of 87% and 81%, respectively. Our test accuracy was 93% with using majority voting on patches of test subjects and sensitivity and specificity were 95% and 90%, respectively.

**Table 1:** Performance of the FW-VF map.

	Accuracy	Sensitivity	Specificity
Cross-validation results (patches from 106 training subject)	85	87	81
Test result using majority voting (30 test subjects)	93	95	90

### Comparison of efficacy of FW-VF classifier with those created from other maps

Table 2 shows the cross-validation result of our CNN using different input maps, including the free water volume fraction (FW-VF), FW corrected fractional anisotropy (FW-FA), FW corrected axial (FW-AX) and radial diffusivity (FW-RAD) maps, a combination of FW-VF and FW-FA (FW-VF + FW-FA), conventional mean diffusivity (MD), fractional anisotropy (FA) maps, and a combination of MD and FA (MD + FA). As seen, FW-VF outperformed the MD and FA maps as well as FW-FA, FW-AX and FW-RAD measures. The combination of FW-VF and FW-FA did not increase the result of free water volume fraction. The results for the test set are provided in Table 3, comparing the FW-VF based classifier with those created using conventional MD and FA maps and free water corrected measures. As seen, free water volume fraction performed better than both MD and FA as well as free water corrected measures.

**Table 2:** The cross-validation (patch-wise) comparison of FW-VF with different input maps.

Input map	Accuracy	Sensitivity	Specificity
FW-VF	85	87	81
FW-FA	81	82	79
FW-AX	74	78	70
FW-RAD	70	75	64
FW-VF + FW-FA	85	88	81
MD	77	75	83
FA	76	74	82
FA + MD	79	78	84

**Table 3:** The test result (subject-wise) comparison of FW-VF with different input maps.

Input map	Accuracy	Sensitivity	Specificity
FW-VF	93	95	90
FW-FA	87	90	80
FW-AX	77	80	70
FW-RAD	74	75	70
FW-VF + FW-FA	93	95	90
MD	83	85	80
FA	80	80	80
FA + MD	83	85	80

### Comparison of CNN with radiomic and texture-based classifiers

**Figure 3:**
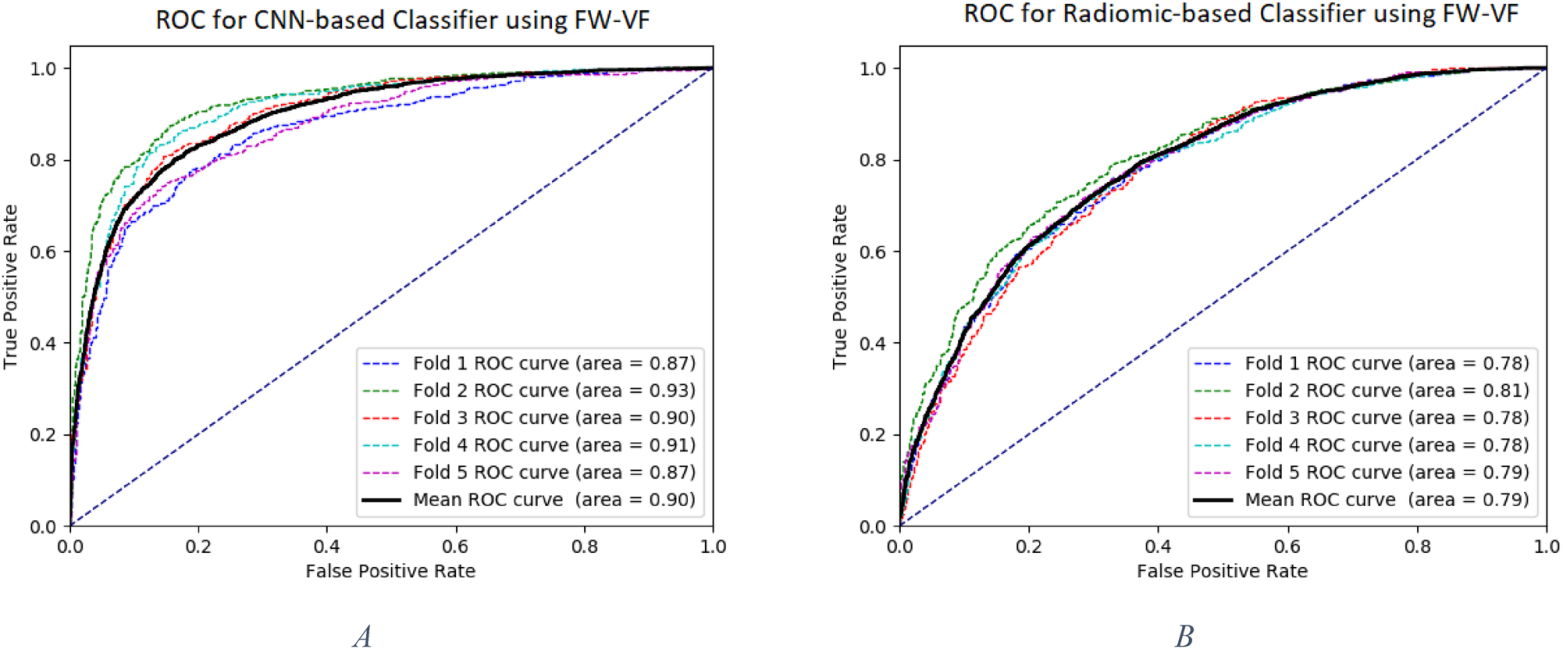
ROC Curves: Comparison between CNN (**A**) and Radiomic features (**B**) on free water volume fraction map. CNN outperformed Radiomics significantly, comparing AUC for mean ROCs (P < 0.0001).

Table 4 shows the cross-validation comparison of the CNN with Gabor and radiomic features in combination with random forest classifiers. ROC curves are also provided in Fig. 3. Area Under the Curves (AUCs) were statistically compared using DeLong et al. method^35^. As seen, CNN outperformed both Gabor and radiomic features significantly. Table 5 shows the result for 30 test subjects calculated by majority voting among patches. CNN outperformed both Gabor and radiomic features with random forest classifiers in the test result as well.

**Table 4:** The cross-validation (patch-wise) comparison of the CNN to Gabor and Radiomic features.

Classification/feature extraction	Accuracy	Sensitivity	Specificity
CNN	85	87	81
Gabor Filters/RF	70	76	67
Radiomic/ RF	76	79	74

**Table 5:** The test result (subject-wise) comparison of the CNN to Gabor and Radiomic features.

Classification/feature extraction	Accuracy	Sensitivity	Specificity
CNN	93	95	90
Gabor Filters/RF	77	80	70
Radiomic/RF	80	80	80

## Discussion

dMRI, especially with the recent advances in multi-compartment modeling, provides an insight into tissue microstructure, that is able to characterize peritumoral area due to the differential water diffusion between pure edema and the one affected by the presence of infiltrative patterns. Free water volume correction applies multicompartment modeling to tease apart tissue fraction and free water volume fraction representative of extracellular water and infiltration. In this paper, we created a classifier for discriminating tumor type based on the characterization of the microstructure of the peritumoral microenvironment, using a DTI-based free water volume fraction map. We used convolutional neural networks to characterize peritumoral area and demonstrated the ability of the CNN and free water volume fraction to distinguish between metastases and glioblastomas.

Our method successfully classified metastatic versus glioblastoma tumors, indicating that there was sufficient information in the microstructure of the peritumoral area to discriminate tumor type and, furthermore, that the information was captured in a DTI-based measurement of extracellular free water content in the peritumoral regions. Metastases and glioblastomas have different underlying microstructures of vasogenic versus infiltrative edema, and we were able to capture the heterogeneity within this area using free water map in conjunction with deep learning. This highlights the importance of multicompartment modeling based approaches on DTI that is able to disentangle the extracellular water from the underlying tissue.

We picked random patches in the peritumoral region of metastases and glioblastomas to train our classifier and achieved 85% accuracy in our cross-validation result. This result indicates that the volume fraction classifier could detect local signatures of glioblastomas which might correspond to such biological processes as infiltration and extracellular matrix damage. It implies that that free water volume fraction could not only discriminate tumor type but also could assess the spatial pattern of extracellular water to characterize the peritumoral area of two tumors and quantify the heterogeneity within the peritumoral region. Those spatial patterns were extracted by a CNN suggesting that the information existed as imperceptible features inside the peritumoral area that was not detected by only using free water map and the best-performing traditional machine learning classifiers. Our CNN, however, was able to capture those features.

Using majority voting on the classification of patches, we achieved 93% accuracy on 30 patients left out from the training. It shows that majority voting among the results of patches boosted the performance and improved the underlying classification accuracy, suggesting that while some patches in the peritumoral area might be individually misclassified, when the peritumoral area was assessed as a whole, such misclassifications tended to be in the minority. This is consistent with the fact that majority of patches in peritumoral area of glioblastomas show infiltrative pattern and vice versa for metastases.

FW-FA had the second-best performance. The fact that FW-VF performed better than FW-FA led us to the conclusion that the most discriminative feature was present in the extracellular water compartment, more than the intracellular or tissue compartment. Free water corrected fractional anisotropy performed better than FW-AX and FW-RAD, suggesting that the relevant feature within the tissue compartment to discriminate heterogeneity in peritumoral area was captured by the overall degree of anisotropy in the diffusion of the tissue compartment, and not axial and radial diffusivity individually.

We compared our FW-VF based classifier with the one trained on traditional FA and MD maps that were the conventional metrics used in previous work to discriminate metastases and glioblastomas^3,6–13^. Glioblastomas exhibit higher diffusivity of water molecules representing destruction of the extracellular matrix ultrastructure by malignant cell infiltration. Thus, mean diffusivity representative of the magnitude of diffusion and fractional anisotropy representative of disorganized diffusion were effective features for the discrimination of glioblastoma and metastases. However, those two maps do not use multicompartment modeling and FW-VF consequently performed better than standard mean diffusivity and fractional anisotropy measures. Our finding showed that the information captured by multicompartment modeling representative of free water could better characterize peritumoral microstructure and discriminate tumor type.

Radiomics and texture features have also been extensively applied in cancer research^36^, most notability in central nervous system malignancies^37^. Radiomics describe intensity, frequency and geometrical characteristics and texture features quantify intrinsic heterogeneous properties from the visual data. Several studies attempted to examine the differentiation of various brain tumor types, including glioblastomas and brain metastases, by Gabor filters as conventional texture features^6,12,32,38–40^ and radiomic features^1,37,41^. The CNN classifier in this paper, was able to capture the pattern of heterogeneity better than the radiomic and Gabor texture features. While Gabor and radiomic features extracted meaningful features like shape, size, histogram, frequency content and other texture-related features, CNN used consecutive layers of convolution and pooling, with each successive layer detecting features at a more abstract level than the layer before. As a result, the CNN extracted imperceptible features which were not constrained to the specific designs of the radiomic or Gabor texture features (shape, size, etc.), and performed better than them.

Our study has a few limitations. First, our dataset was unbalanced in terms of the number of patients. However, we constructed a balanced sample of patches for metastases and glioblastomas which included ~ 6300 patches (~ 3000 metastases and ~ 3300 glioblastomas) from the 106 training subjects to make a balanced training set for our classifier. We also reported sensitivity and specificity for metastases and glioblastomas in which our sensitivity determined the number of glioblastoma patients retrieved correctly and vice versa for specificity and metastases. Second, our data was acquired at a single institution, however, it included different acquisition protocols and we used an independent test cohort to make sure that our method is generalizable.

Our study opens horizons for future work. First, majority of our metastases class were from lung cancer followed by breast and melanoma and our glioblastoma patients all were IDH1-wildtype. While our sample size after breaking down by original cancer type and IDH mutations was not large enough to do analyses on these subdivisions, in the future, our imaging signature can be applied to imaging data of different metastases and molecular sub-types of tumors. Second, our model only used extracellular water differences between metastases and glioblastomas to diagnose tumor types. Our contribution was to introduce new imaging signature based on extracellular water differences in peritumoral area which outperforms other diffusion measures like mean diffusivity and fractional anisotropy. In future, it can potentially be improved by integrating multi-model imaging data (such as structural imaging, other diffusion tensor measures, or perfusion imaging).

We proposed a deep learning-based approach for discriminating brain tumor types using patterns of free water volume fraction map in the peritumoral microenvironment. Free water volume fraction emerged as an encouraging tool for better characterization of the peritumoral edema and tumor type distinction. We achieved superior performance with respect to pattern recognition methods and conventional MD and FA approaches. This will encourage existing frameworks using DTI measures for characterizing peritumoral environment to replace their maps with free water volume fraction maps and can further be combined with other modalities like structural MRI to provide deeper insight into tissue microstructure and the detection of other type of tumors.

## Conclusion

We propose an approach based on free water volume fraction map to characterize the peritumoral microenvironment. Due to differences in microstructural patterns of extracellular water in peritumoral regions reflecting the characteristics of the underlying tumor, free water volume fraction is a successful compartment for discriminating tumor types with CNN. It reflects pattern of extracellular water and infiltration which is not visible in conventional imaging metrics and therefore reveals important information in combination with CNN. Our method provides further non-overlapping information about the peritumoral microstructure that can be used to characterize peritumoral area and discriminate tumor type. This novel approach potentially could complement, or even replace, standard DTI indices, such as fractional anisotropy, or mean diffusivity to provide an integrated, biologically relevant characterization of the peritumoral microenvironment.

## Supplementary Information

Supplementary Information.

## Data Availability

The datasets generated during the current study are not publicly available due to the IRB requirements of the Hospital of University of Pennsylvania. The datasets can be made available on request to the corresponding author, after required data transfer and IRB paperwork is completed.
