# Supplementary material for: Distinct tumor signatures using deep learning-based characterization of the peritumoral microenvironment in glioblastomas and brain metastases
Source: Sci Rep. 2021 Jul 14;11:14469. doi: 10.1038/s41598-021-93804-6 (PMC8280204; doi:10.1038/s41598-021-93804-6)

Distinct Tumor Signatures using Deep Learning-based Characterization of the Peritumoral Microenvironment in Glioblastomas and Brain Metastases

Zahra Riahi Samani[1], Drew Parker[1], Ronald Wolf [2], Wes Hodges[3], Steven Brem[2], Ragini Verma[1]*

1) Zahra.riahisamani@pennmedicine.upenn.edu, William.parker@pennmedicine.upenn.edu, Ragini@pennmedicine.upenn.edu
Diffusion and Connectomics in Precision Healthcare Research Lab (DiCIPHR), Department of Radiology, University of Pennsylvania, Philadelphia, PA

2)Ronald.Wolf@pennmedicine.upenn.edu, Steven.brem@pennmedicine.upenn.edu
Department of Radiology, Department of Neurosurgery, University of Pennsylvania, Philadelphia, PA

3) Wes.hodges@synaptivemedical.com,
Founder at Synaptive Medical, Toronto, Ontario, Canada

Correspondence:
Ragini Verma
Ragini@pennmedicine.upenn.edu

S.1. Details of CNN architecture:

Our CNN contained three pairs of convolution layer, followed by a max pooling layer, a global average pooling layer and two fully-connected layers. The hyper-parameters of CNN were weight decay $5 \times 10{-}5$, momentum 0.9, initial learning rate $10{-}4$. The CNN model was trained with patches that were automatically extracted from peritumoral area of metastases and glioblastomas. We picked (32, 32) patches which were input to the CNN, with (3*3) kernels and channel size of (32, 64, 128), followed by a max pooling layer and a global average pooling. As our patch size was small, we did not add more layers and we only used one pooling layer.

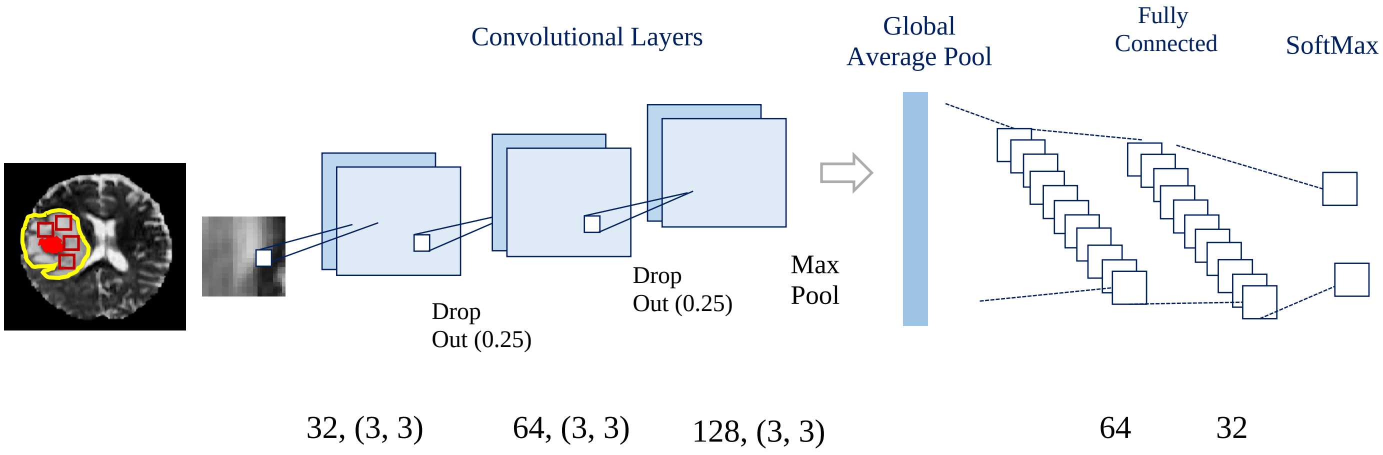

Supplement: Supplementary file 1 — Supplementary Information. [file 41598_2021_93804_MOESM1_ESM.pdf]
